# New Anthropometry-Based Formulae to Predict 24 h Sodium Excretion from Spot Urine

**DOI:** 10.3390/nu17203284

**Published:** 2025-10-20

**Authors:** Martina Zandonà, Karin Holzner, Maria Luisa Garo, Rosaria Del Giorno, Luca Gabutti

**Affiliations:** 1 Family Medicine Service, Ente Ospedaliero Cantonale, 6900 Lugano, Switzerland; 2 Faculty of Biomedicine, University of Southern Switzerland, 6900 Lugano, Switzerland; karin.holzner@usi.ch; 3 Biostatistic Unit, Mathsly Research, 00128 Rome, Italy; marilu.garo@mathsly.it; 4 Angiology Service, Ente Ospedaliero Cantonale, 6500 Bellinzona, Switzerland; rosaria.delgiorno@eoc.ch; 5 Family Medicine Institute, Università della Svizzera Italiana, 6900 Lugano, Switzerland

**Keywords:** 24 h urine, sodium excretion, salt intake, spot urine, first morning urine, prediction formulae, Swiss population

## Abstract

**Background:** Cardiovascular diseases are the leading cause of death globally, with hypertension and high sodium intake being major contributors. Accurate estimation of sodium intake is essential, but 24 h urine collection, the gold standard, is cumbersome and impractical for routine clinical use. Existing spot urine-based prediction formulae lack accuracy at the individual and population level. **Objective:** To develop and validate population-specific formulas for estimating 24 h urinary sodium excretion from spot urine samples using data from a representative Swiss adult population. **Methods:** Models with and without urea and potassium were developed incorporating age, sex, and anthropometry-based, population-specific, estimated urinary creatinine excretion values. Data quality was rigorously controlled, and model performance was compared to the INTERSALT, Kawasaki, and Tanaka formulae and to a nocturnal timed urine collection used to calculate hourly creatinine excretion. **Results:** Models based on first morning urine demonstrated improved accuracy (AUCs: Swiss anthropometric model 0.85 (95% CI: 0.80–0.90), Swiss anthropometric model with urea 0.86 (95% CI: 0.81–0.91)) and lower bias (−5.5 mmol/24 h for the Swiss anthropometric model and −2.86 mmol/24 h for the Swiss anthropometric model with urea) compared to existing equations. Performance was consistent across clinically relevant sodium intake thresholds and the models were therefore suitable for use in clinical settings. A timed nocturnal urine collection further improves accuracy. **Conclusions:** These new simple and reliable formulae provide a promising and practical tool for estimating sodium intake from first morning urine spot in adult European populations, and are potentially applicable in clinical settings.

## 1. Introduction

Cardiovascular diseases (CVDs), including coronary disease and stroke, are the leading cause of death globally [1]. Hypertension is one of the main pathophysiological causes of CVDs [2] and plays a major role in the development and progression of chronic kidney disease (CKD) [3], left ventricular hypertrophy, and increased arterial stiffness [4]. Salt consumption correlates with the risk of developing high blood pressure values [5,6,7], and in addition promotes oxidative stress and renal fibrosis, which may contribute to impaired endothelial function and, in turn, cause CKD progression [8].

Due to these associations, several international guidelines suggest reducing salt consumption to less than 5 g (90 mmol of NaCl) per day, equivalent to approximately 2 g of sodium [9,10].

Despite these recommendations, a 2018 review showed that salt intake in developed countries ranged from 6.7 g/day to 10.7 g/day [11]. The Swiss Salt Study 2, which took place between 2022 and 2023 in Switzerland, showed a salt intake between 5.1 and 12.3 g/day in the Swiss population [12]. Given the difficulty of meeting recommended levels of salt intake, almost all WHO Member States have made a political commitment to reducing it, and 27% have already implemented mandatory policies [13].

Several methods have been applied to estimate sodium intake at the population level. Questionnaires are inexpensive and relatively easy to administer but are prone to error and tend to underestimate true dietary sodium intake [14], while 24 h urine collection with determination of sodium content, considering that approximately 90% of ingested sodium is excreted through urine, has become the gold standard [15,16,17]. However, collecting the urine is time-consuming, difficult to perform accurately, and places a high burden on participants. Incomplete or incorrect collections, poor sample storage, or incomplete bladder emptying can in fact lead to significant errors. As a result, timed urine collections are difficult to apply in both clinical routine and large-scale epidemiological studies [18].

Several approaches can be implemented to assess the completeness of 24 h urine collections, including self-reporting procedures and the use of models to estimate 24 h urinary creatinine excretion [19].

A new anthropometry-based, age- and sex-specific reference formula to estimate 24 h urinary creatinine excretion has recently been developed based on the adult Swiss population [20]. The study population represents all the different language regions and cultures of Switzerland (Italian, French, German, and Romansh) and can be considered a valid reference for the Central European population.

Given the critical issues related to 24 h collection, it is important to explore simpler and more feasible ways to estimate dietary sodium intake. Methods based on spot urine samples (where urinary creatinine concentration is used to correct for the influence of urinary volume and water excretion on sodium concentration), have been investigated as practical alternatives [21], and several related equations have been developed to estimate 24 h sodium excretion. The most used are the Tanaka [22], INTERSALT [23], and Kawasaki [24] equations. All of these however demonstrate systematic bias, with overestimation of sodium intake at low levels of sodium excretion and underestimation of it at high levels [21]. Furthermore, there is no clear evidence regarding which equation is the most reliable, and accuracy varies across populations [25].

Most validation studies rely on a single 24 h collection or dietary assessment tools, while validation should ideally be based on repeated 24 h urine collections or controlled feeding studies with known sodium intake [26].

The International Consortium for Quality Research on Dietary Sodium/Salt has therefore concluded that more high-quality validation studies are needed to assess the validity of spot urine sampling for population-level sodium intake [26].

Several studies have in the meantime shown the poor performance of the available equations for estimating sodium intake at the individual level [27].

One reason why spot urine may not be representative of 24 h urine is that sodium excretion follows a circadian rhythm, which appears to be linked to the behavior of blood pressure [28]. Blood pressure typically decreases by 10–20% at night compared to daytime values, a phenomenon referred to as “dipping” [29]. In healthy individuals, sodium excretion is higher during the day than at night, while individuals with a non-dipping blood pressure pattern tend to excrete a higher fraction of sodium during the night. The non-dipping pattern is in turn associated with increased risk of CVD, renal disease, and other end-organ damage such as left ventricular hypertrophy, microalbuminuria, and cerebrovascular disease [30]. The absence of a dipping pattern can be related, among other causes, to high sodium intake and/or salt sensitivity [31,32]. It has also been suggested that it could result from a reduced capacity to excrete sodium during the daytime, leading to compensatory excretion during the night [31,33,34].

In summary, the reasons behind the inaccuracy of the formulae used to predict 24 h urinary sodium excretion based on spot urine could be the variation in sodium excretion related to its circadian rhythm, the use of a potentially inadequate urine collection for model construction, and the need for specific population-based prediction formulae to estimate 24 h urinary creatinine excretion.

We attempt to resolve these critical issues by using a formula to predict 24 h urinary creatinine excretion based on an epidemiological study representative of the Swiss population and, using the same strategy, by excluding potentially inadequate 24 h urine collections. Last but not least, we will test in our prediction models the added value to use the urinary concentrations of potassium (which tends to be inversely associated with sodium intake) [35] and urea (proportional to protein intake and associated with sodium intake) [36,37], and a timed nocturnal urine collection to assess instead of estimating hourly creatinine excretion.

Specifically, we assess the performance of commonly used equations for estimating 24 h sodium excretion based on spot urine samples within our study population, and develop and validate new estimation formulae based on our epidemiological data, which has been rigorously checked for quality, using a population-specific anthropometry-based model. This approach may provide a more accurate and physiologically informed method for estimating sodium intake in future research and clinical practice.

## 2. Materials and Methods

### 2.1. Study Design and Population

This study is based on data from the Ticino Epidemiological Stiffness Study (TEST study), a cross-sectional study conducted between 2017 and 2018 in Southern Switzerland [38]. The participants were selected based on a list provided by the Swiss Federal Statistical Department of the resident population in Ticino. A simple random sampling method considering residence, gender, and age group was used. Of the 1400 residents invited, 1202 (86%) agreed to participate by completing the study protocol. All participants provided written informed consent. The study was carried out in accordance with the 1964 Helsinki Declaration and approved by the local Swiss ethics committee (CE 3115-2016-01718). After cleaning the database by eliminating subjects with incomplete or implausible values, the final analysis included data from 811 adult participants who underwent a standardized assessment of 24 h urinary sodium excretion. Separately gathered 24 h diurnal and nocturnal urine collections were obtained from all participants, along with anthropometric and demographic data. For model development, the data set was randomly divided into a training cohort (*n* = 574, 70.8%) and a test cohort (*n* = 237, 29.2%), used for model training and internal validation, respectively.

### 2.2. Data Collection Procedures

All study subjects participated in two clinical visits at the research unit of the Internal Medicine Department of the Regional Hospital of Bellinzona (Switzerland). Each participant was given a standardized questionnaire covering sociodemographic characteristics, comorbidities, traditional cardiovascular risk factors, eating habits, and detailed drug anamnesis. Participants were also investigated regarding their usual diet. Height (cm), weight (kg), body mass index (BMI; kg/m^2^), waist circumference (cm), hip circumference (cm), and neck circumference (cm) were measured. Fasting blood samples were drawn and analyzed for lipid profile, serum glucose, HbA1c, creatinine, and electrolytes. Blood pressure (BP) was measured in the clinic with a validated automatic oscillometric device (Dinamap model Pro 100, Critikon, Tampa, FL, USA) using a standardized procedure. After the first session, participants underwent 24 h ambulatory blood pressure measurement (ABPM) using a Mobil-O-Graph device, which recorded every 30 min during the day and hourly during the night.

On the same day as the ABPM, 24 h urine samples were collected. Two distinct urine collections were performed: one during the day and one at night. In the morning, study participants were given thorough instructions outlining the collection process when they visited the research unit. Two 2 L urine collection containers labeled “daytime urine” and “nighttime urine,” as well as a plastic beaker for transferring urine, were provided during the initial visit. Day and night were defined based on each participant’s self-reported bedtime and wake-up time. The first urine of the morning was added to the nighttime collection. Each participant was also given a urine collection diary, in which they were asked to record (i) the hour at which urine collection began during the day, (ii) the hour at which it began at night, and (iii) the hour of the most recent urine sample. All urine was collected over the following 24 h period. Afterward, participants returned to the hospital and delivered the urine to the Biochemistry Laboratory of Ente Ospedaliero Cantonale, Regional Hospital of Bellinzona. Concentrations of urinary sodium, potassium, creatinine, and urea were measured using a Roche Cobas 8000 analyzer (Roche, Basel, Switzerland).

### 2.3. Data Preprocessing and Cleaning

**Figure 1:**
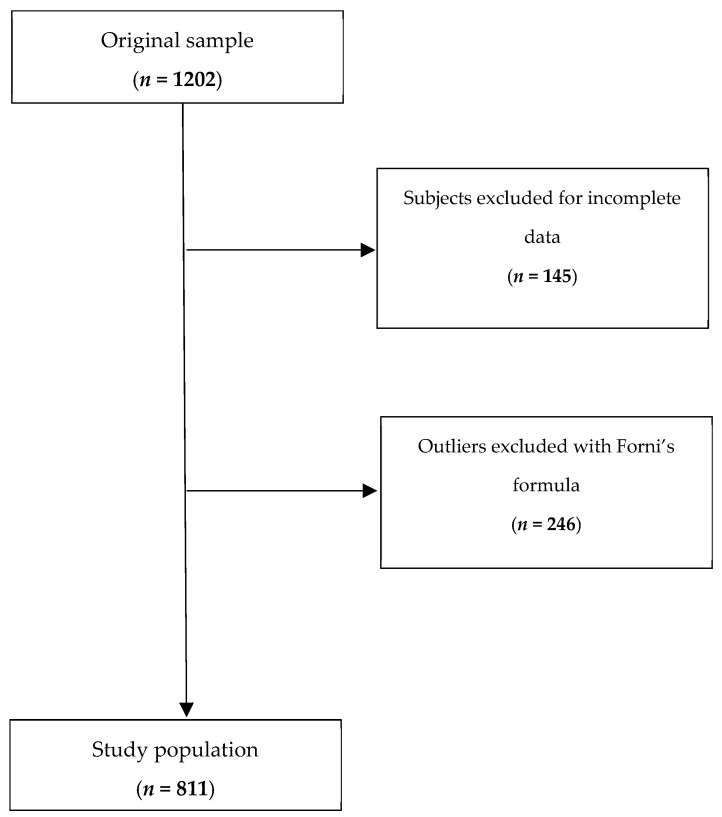
Flowchart showing the participant selection procedure.

Prior to model development, the entire data set, which originally consisted of 1202 observations, underwent extensive preprocessing to ensure data integrity and analytical robustness. The process of data cleaning was performed in two main steps: (1) identification of raw data errors and inconsistencies in demographic, anthropometric, and biochemical variables, with the exclusion of 145 subjects, and (2) detection and exclusion of outliers, with the exclusion of 246 subjects (Figure 1). For this last step, as a preliminary measure, Forni’s formula was applied to estimate 24 h creatinine excretion based on individual anthropometric characteristics. The difference (delta) between the estimated and observed creatinine values was then calculated for each subject. Extreme values were then removed using a centile-based approach excluding observations where the delta exceeded the 1st or 99th percentile. We removed the extremes because they were likely expressions of inadequate urine collections (24 h urine collection often being unreliable due to the complexity of the procedure) [39]. The aim of the procedure was to minimize the influence of outliers that could bias the regression estimates while preserving the majority of the data set. The statistical approach used is a robust non-parametric technique that does not assume a particular distribution and is suitable for working with biological data that are prone to variability and occasional measurement errors.

### 2.4. Model Development

Descriptive statistics were calculated and presented as mean ± standard deviation [median and interquartile range] for continuous variables and absolute frequencies with percentages for categorical variables. The data set was then split into a training set and a test set. The comparability between the two subsets was then assessed using the Mann–Whitney U test for continuous variables and the chi-square test for categorical variables. The models were developed after verifying that there were no significant differences between the subsets.

Four multivariable linear regression models were trained on the training cohort to predict 24 h sodium excretion from the urine data. The decision to use this modeling approach was based on an extensive review of previous similar validation studies, on the methodological limitations they highlighted in the prediction equations [22,23,24], and considering the high misclassification rate when applied at the individual level [40]. Therefore, we decided to investigate customized multivariable regression models incorporating both anthropometric and biochemical features. Prior to model specification, we tested the relationship between the predictors and the outcome for potential non-linearity. To do this, we used exploratory plots to assess non-linear trends, followed by statistical tests for non-linearity of the regression residuals. Each model was developed in two parallel versions—one based on first morning urine with estimated creatinine excretion (Forni’s formula) and the other based on timed nocturnal urine with measured creatinine excretion—to assess which sample type provides better predictive performance in our population.

Swiss anthropometric model with potassium (SAMK): first morning or timed nocturnal urine, age, weight, height, and urinary potassium concentration. The final models derived from the first morning and the timed nocturnal urine, respectively, had the following regression equation (β-coefficients from the training set):

Na24h (mmol/die) = 67.75 + 0.54 × *Na_Night/Creat_Night* × *Estimated Creatinine* (mmol/L) + 0.05 × *Potassium Night* (mmol/L) + 0.52 × *Weight* (kg) − 0.06 × *Height* (cm) − 0.52 × *Age* (years)

Na24h (mmol/die) = 63.11 + 0.58 × *Hourly Na concentration* (mmol/L) × *24* h + 0.03 × *Potassium Night* (mmol/L) + 0.46 × *Weight* (kg) + 0.04 × *Height* (cm) − 0.51 × *Age* (years)

Swiss anthropometric model (SAM): built on the anthropometric model excluding potassium. The corresponding regression formulae were

Na24h (mmol/die) = 59.83 + 0.57 × (*Na_Night/Creat_Night* × *Observed Creatinine*) + 0.47 × *Weight* (kg) − 0.01 × *Height* (cm) − 0.44 × *Age* (years)

Na24h (mmol/die) = 53.02 + 0.59 × *Hourly Na concentration* (mmol/L) × 24 h + 0.41 × *Weight* (kg) + 0.03 × *Height* (cm) − 0.49 × *Age* (years)

Swiss anthropometric model with urea and potassium (SAMUK): built on the anthropometric model by adding urinary urea concentration (β-coefficients from the training set). The corresponding regression formulae were

Na24h (mmol/die) = 67.19 + 0.56 × *Na_Night/Creat_Night* × *Estimated Creatinine* (mmol/L) + 0.05 × *Urea Night* (mmol/L) − 0.18 × *Potassium Night* (mmol/L) + 0.44 × *Weight* (kg) − 0.09 × *Height* (cm) − 0.44 × *Age* (years)

Na24h (mmol/die) *=* 61.89 *+* 0.59 × *Hourly Na concentration* (mmol/L) × 24 h + 0.04 × *Urea Night* (mmol/L) − 0.18 × *Potassium Night* (mmol/L) + 0.39 × *Weight* (kg) − 0.06 × *Height* (cm) − 0.44 × *Age* (years)

Swiss anthropometric model with urea (SAMU): identical to model 3, but without potassium to improve the parsimony of the model and its feasibility in resource-poor environments (β-coefficients from the training set)

Na24h (mmol/die) = 69.56 + 0.56 × *Na Night/Creat Night* × *Estimated Creatinine* (mmol/L) + 0.03 × *Urea Night* (mmol/L) + 0.45 × *Weight* (kg) − 0.11 × *Height* (cm) − 0.47 × *Age* (years)

Na24h (mmol/die) = 64.13 + 0.59 × *Hourly Na concentration* (mmol/L) × 24 h + 0.03 × *Urea Night* (mmol/L) + 0.39 × *Weight* (kg) − 0.08 × *Height* (cm) − 0.47 × *Age* (years)

where Na24h is the predicted 24 h sodium excretion (mmol/die) and all biochemical concentrations were derived from either first morning or timed nocturnal urine samples.

### 2.5. Model Evaluation

The performance of the models was evaluated in the test set using several statistical metrics: (1) the coefficient of determination (R^2^), which quantifies the proportion of variance in the observed sodium excretion explained by the model; (2) the root mean squared error (RMSE), which is used to assess the prediction error and penalize larger deviations more heavily; (3) the mean deviation, which is calculated as the average difference between predicted and observed values to detect systematic over- or underestimation; (4) the proportional bias, to determine whether prediction errors vary with the level of sodium excretion; and (5) the mean relative error ± standard deviation, which expresses prediction accuracy as a percentage. In addition, the proportion of individuals with a relative error greater than 40% was calculated to identify clinically significant inaccuracies.

Agreement between predicted and observed values was also assessed using Bland–Altman plots, with limits of agreement set at ±1.96 standard deviations from the mean difference. An acceptable clinical margin was defined as ±10 mmol/day.

To assess discriminatory ability, the area (AUC) under the receiver operating characteristic curve (ROC; plotting the true positive rate on the *y*-axis against the false positive rate on the *x*-axis) was calculated for sodium excretion thresholds of 85, 150, and 200 mmol/day, corresponding to low (WHO recommendation), medium (Swiss population average), and high sodium intake, respectively. The model AUCs were compared with those of the standard equations (Kawasaki, Tanaka, and INTERSALT).

Calibration was examined by regressing the observed sodium excretion on the predicted values in the test set. An ideal calibration was characterized by an intercept close to zero and a slope close to one. Calibration metrics were derived via ordinary least squares regression. All analyzes were performed using STATA version 19 (StataCorp, College Station, TX, USA).

## 3. Results

A total of 811 participants were included in the analysis (training set: *n* = 574; test set: *n* = 237). The baseline characteristics were balanced in the two groups. There were no significant differences in age, sex distribution, BMI, or 24 h urinary sodium excretion (*p* > 0.05 for all comparisons; Table 1). Concerning the prevalence of hypertension in the selected population, office blood pressure severity classification indicated that 20.1% of the participants had Grade 1 (systolic 140–159 mmHg and/or diastolic 90–99 mmHg); 6.4% Grade 2 (systolic BP between 160 and 179 mmHg and/or diastolic between 100 and 109 mmHg), and 1.2% Grade 3 hypertension (≥180/110 mmHg). Ambulatory blood pressure monitoring (ABPM) revealed that 16.4% had 24 h systolic hypertension (≥130 mmHg), whereas 24.7% presented with 24 h diastolic hypertension (≥80 mmHg). Daytime systolic hypertension (≥135 mmHg) was detected in 14.1%, while nighttime systolic hypertension (≥120 mmHg) was observed in 21.7%. Among all subjects, 12.1% were undergoing antihypertensive therapy (54%, 34%, and 12% with 1, 2, or 3 or more drugs, respectively). As for renal dysfunction, 1.7% of the participants had chronic kidney disease (CKD) stage 3 or greater. The remaining characteristics of the population (including waist circumference, hip and neck circumferences, lipid profile, serum glucose, HbA1c, creatinine, and electrolytes) have been published previously [41,42].

Four predictive models for estimating 24 h urinary sodium excretion, using, respectively, the first morning and timed nocturnal urine samples, were evaluated. Both were built using anthropometric data with or without urinary urea concentration and with or without urinary potassium concentration, and were validated.

In the test set using first morning urine (Table 2), SAMK achieved an R^2^ of 0.52 with an RMSE of 38.06 mmol/24 h and a mean difference of −5.5 mmol/24 h (SD: 37.99). The same model without potassium achieved an R^2^ of 0.53 with an RMSE of 37.99 mmol/24 h and a mean difference of 4.23 mmol/24 h (SD: 37.91). SAMUK performed slightly better (R^2^ = 0.54, RMSE = 37.38) and had a lower mean deviation (−2.86 mmol/24 h). The mean relative error was minimal for all three models (3.65%, 4.49%, and 5.54%, respectively), and the prediction error was within ±40% for about 78% of the subjects. The fourth model—SAMU—had an R^2^ = 0.53, an RSME of 37.48, and a mean difference of −5.01 mmol/24 h (SD: 27.98), with the predicted error within ±40% for about 80% of subjects. All models showed a proportional bias with a negative slope (approximately −0.4), indicating a moderate underestimation at higher sodium levels. Training set multivariate regression model results are reported in Supplementary Materials, Tables S1 and S2.

**Figure 2:**
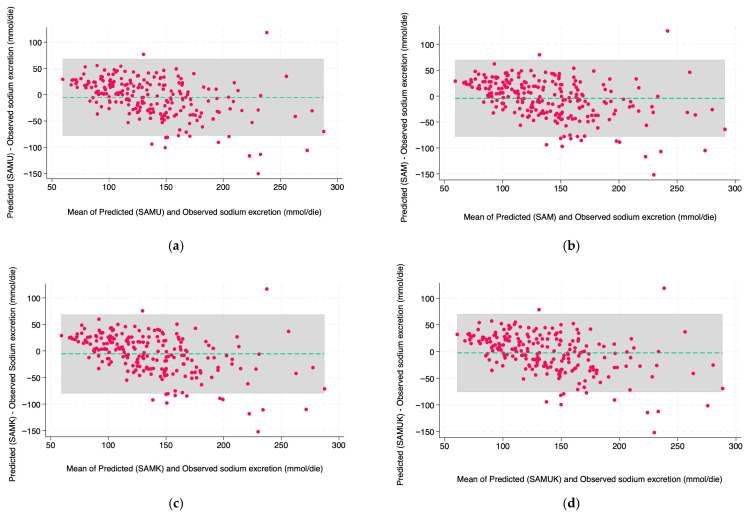
Agreement between observed and predicted 24 h sodium excretion using daytime spot urine samples. Each panel shows results for: (**a**) SAMK, (**b**) SAM, (**c**) SAMUK, and (**d**) SAMU. The central dashed line indicates the mean difference (bias), and the gray shaded area represent the 95% limits of agreement (±1.96 SD).

The agreement between predicted and observed sodium excretion using first morning urine was assessed using Bland–Altman plots (Figure 2a–d). For SAMUK (Figure 2c), the mean difference was −2.86 mmol/24 h, with the limits of agreement lying approximately between −76 and +70 mmol/24 h. The plot showed a narrow spread of differences around the mean, indicating a consistent prediction across the range of sodium levels. No systematic trend in differences was observed, although there was a slight tendency to underestimate at higher excretion values (>200 mmol/24 h).

SAMU (Figure 2d) showed a similarly good agreement, with a mean deviation of −5.01 mmol/24 h and limits of agreement of about −78 to +68 mmol/24 h. The distribution of residuals was symmetrical, and the overall agreement was comparable to SAMUK, confirming that the exclusion of potassium does not significantly affect accuracy. Both plots showed that over 95% of the differences were within the calculated limits of agreement, which corresponds to acceptable clinical performance. These visual analyses confirm the minimal systematic error and limited scatter associated with SAMU and SAMUK.

**Figure 3:**
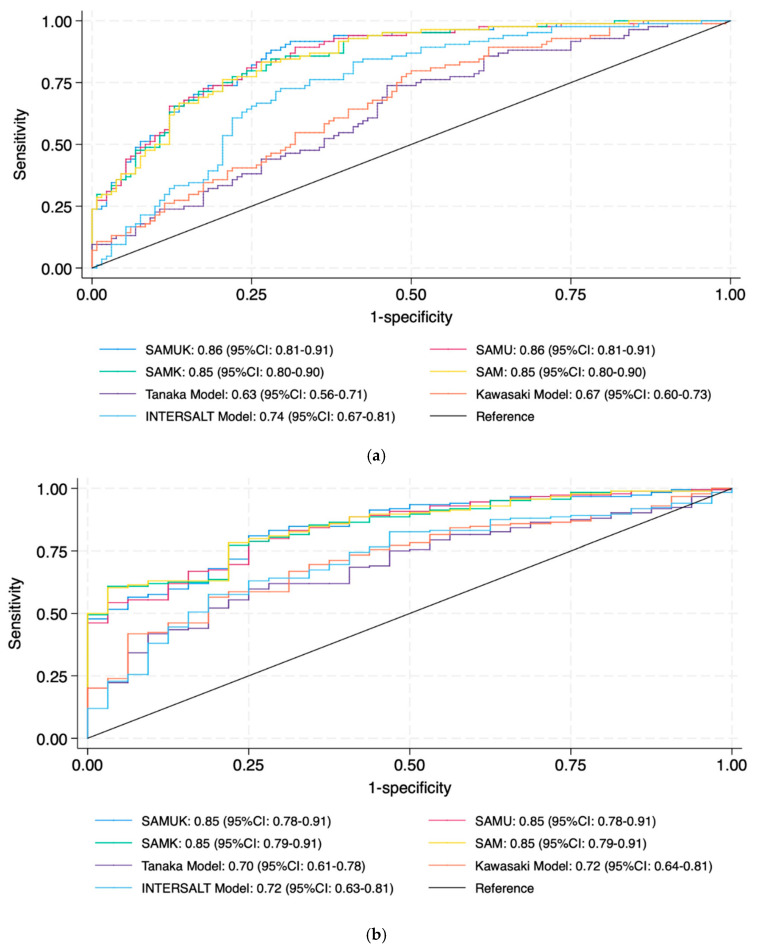

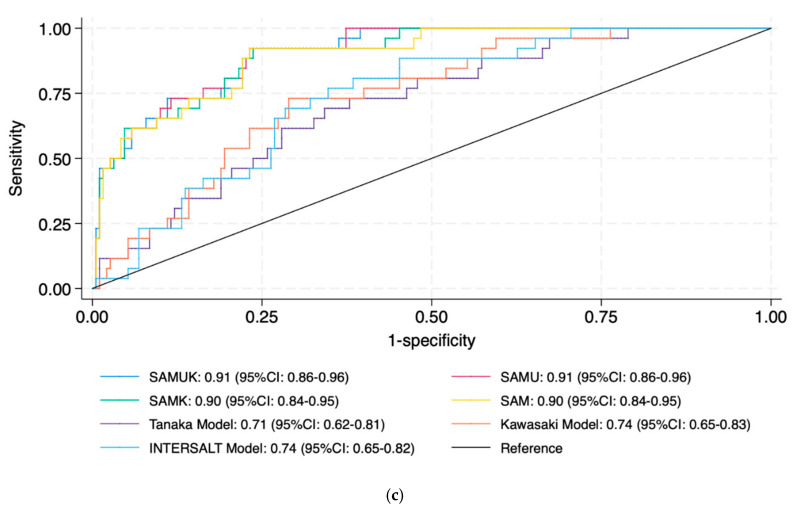
Receiver operating characteristic curves for the four predictive models using first morning urine, evaluated at sodium excretion thresholds of: (**a**) 150 mmol/day, (**b**) 85 mmol/day, and (**c**) 200 mmol/day.

The analyses based on ROC curves corresponding, respectively, to thresholds of 150, 85, and 200 mmol/24 h, showed optimal discriminatory power (Figure 3a–c). For the threshold of 150 mmol (Figure 3a), the AUCs were as follows: SAMUK 0.86 (95% CI: 0.81–0.91), SAMU 0.86 (95% CI: 0.81–0.91), SAMK 0.85 (95% CI: 0.80–0.90), and SAM 0.85 (95% CI: 0.80–0.90), indicating excellent discriminatory power. These performances were clearly superior to those of the traditional formulae: Tanaka (AUC = 0.63, 95% CI: 0.56–0.71), Kawasaki (AUC = 0.67, 95% CI: 0.60–0.73), and INTERSALT (AUC = 0.74, 95% CI: 0.67–0.81). At the lower threshold of 85 mmol/24 h (Figure 3b), the four models again showed strong discrimination: the SAMUK and SAMU both had AUCs of 0.85 (95% CI: 0.78–0.91), and both SAM and SAMK matched these results with an AUC of 0.85 (95% CI: 0.79–0.91). In contrast, the Tanaka, Kawasaki, and INTERSALT formulae showed lower AUCs (0.70, 0.72, and 0.72, respectively), with upper 95% confidence limits not exceeding 0.81, suggesting suboptimal performance at this threshold. At the highest threshold (200 mmol/24 h, Figure 3c), all four models maintained high accuracy, each achieving an AUC higher than 0.90 (SAMUK: 0.91, 95% CI: 0.86–0.96; SAMU: 0.91, 95% CI: 0.86–0.96; SAMK: 0.90, 95% CI: 0.84–0.95; and SAM: 0.90, 95% CI: 0.84–0.95). These values emphasize the robustness of the models across a broad clinical spectrum. The traditional formulae again underperformed—Tanaka (AUC = 0.71, 95% CI: 0.62–0.81), Kawasaki (0.74, 95% CI: 0.65–0.83), and INTERSALT (0.74, 95% CI: 0.65–0.82)—highlighting the limited utility of existing methods for detecting high sodium excretion.

SAMK showed a calibration slope of 1.035 (95% CI: 0.901–1.169) and an intercept of 0.81 (*p* = 0.933), indicating good agreement between predicted and observed values and the absence of significant bias. Similarly, SAM yielded a calibration slope of 0.998 (95% CI: 0.877–1.120) and an intercept of 4.44 (*p* = 0.615), suggesting good linear agreement, although the intercept was numerically higher. The SAMUK model yielded a slope of 1.032 (95% CI: 0.902–1.161) with an intercept of −1.47 (*p* = 0.875), while the SAMU yielded a slope of 1.031 (95% CI: 0.902–1.161) and an intercept of 0.77 (*p* = 0.934). In three out of four models, the intercepts were statistically indistinguishable from zero and the slopes were close to 1, confirming excellent calibration. These results were further confirmed by R^2^ values between 0.52 and 0.54 and calibration plots showing strong agreement with the line of identity.

### Timed Nocturnal Urine

When using timed nocturnal urine samples (Table 3), all models also performed well. SAMU and SAMUK achieved an R^2^ of 0.58 with an RMSE of 35.52 and 35.54 and a bias of −2.76 and −2.60 mmol/24 h, respectively. Although SAMK showed a higher bias (+10.37 mmol/24 h), it had comparable overall error metrics (R^2^ = 0.57, RMSE = 36.12). Notably, SAM showed a lower mean difference (–3.88 ± 35.84 mmol/24 h), indicating improved accuracy. Relative error distributions remained within acceptable clinical margins, and all models maintained over 70% of predictions within ±40% relative error. Complete results related to the training set are reported in Supplementary Materials Table S2.

Analysis of the ROC curve for nocturnal urine samples underpinned the performance of the models at clinically relevant thresholds (Table 4). At a threshold of 150 mmol/24 h, the AUCs were: SAMUK 0.88 (95% CI: 0.84–0.93), SAMU 0.89 (95% CI: 0.84–0.93), anthropometry model 0.88 (95% CI: 0.84–0.93), and SAM 0.88 (95% CI: 0.83–0.93). These values reflect those observed with first morning urine, indicating the robustness of the model regardless of the timing of the collection. At the lower threshold of 85 mmol/24 h, all models again achieved high discrimination: the SAMUK and the SAMU both had AUCs of 0.87 (95% CI: 0.81–0.92), and SAMK also achieved 0.87 (95% CI: 0.82–0.92). This consistency underlines the suitability of all four models for detecting low sodium excretion. At the upper clinical threshold of 200 mmol/24 h, the models maintained their excellent classification accuracy: SAMUK 0.92 (95% CI: 0.87–0.96), SAMU 0.92 (95% CI: 0.88–0.97), and SAM and SAMK 0.92 (95% CI: 0.87–0.96). These performances were significantly better than those of the traditional reference equations, which in turn showed lower AUCs: Tanaka (0.71, 95% CI: 0.62–0.81), Kawasaki (0.74, 95% CI: 0.65–0.83), and INTERSALT (0.74, 95% CI: 0.65–0.82).

SAMK achieved a calibration slope of 1.014 (95% CI: 0.895–1.134) with an intercept of −12.53 (*p* = 0.185), suggesting good linear agreement but a numerically high intercept. A better result emerged with SAM, in which the calibration slope was 1.01 (95% CI: 0.90–1.12) with an intercept of 2.10 (*p* = 0.796). Although not statistically significant, this may indicate a slight tendency to underestimate sodium excretion among individuals with lower observed values. SAMUK demonstrated a slope of 1.031 (95% CI: 0.913–1.149) and an intercept of −1.60 (*p* = 0.852), confirming near-ideal calibration. Similarly SAMU produced a slope of 1.029 (95% CI: 0.911–1.147) and an intercept of −1.22 (*p* = 0.887).

## 4. Discussion

The primary goal of this study was to develop a simplified, population-based formula to estimate 24 h sodium excretion from a spot urine sample to be used in epidemiological studies and in clinical settings. The established formulae such as INTERSALT, Kawasaki, and Tanaka demonstrate a limited applicability due to low reproducibility and poor accuracy. Our results confirm that our formulae, based on anthropometric data, potassium, and/or urea, perform better than existing equations across the selected sodium excretion thresholds. The Bland–Altman plots showed that data are not systematically skewed, the observed biases are not clinically significant (−5.5 mmol/24 h for SAMK and −2.86 mmol/24 h for SAMUK), and that the overall performance supports the models’ reliability.

To further assess the clinical applicability of the models, we used ROC curves with three distinct sodium excretion cut-off values: 85 mmol/24 h (recommended by the WHO) [43], 150 mmol/24 h (representing the Swiss population average) [12], and 200 mmol/24 h (arbitrarily defined as very high sodium intake). These cut-offs allowed us to evaluate model performance across clinically and epidemiologically relevant benchmarks. For all three thresholds, our models showed superior sensitivity and specificity when compared with INTERSALT, Kawasaki, and Tanaka. However, there was a clinically non-significant underestimation of sodium intake in the high sodium excretion group.

We point out that our motivation to develop a new equation to estimate 24 h sodium excretion from a spot urine sample came from the lack of a simple and reliable procedure that can be applied in the clinical setting.

Our models are easy to apply and suitable for clinical settings, where simplicity, low cost, and reasonable accuracy are important. This reinforces their potential not only for research, but also for practical use such as dietary counseling, public health screening, and monitoring of adherence to salt-reduction interventions.

Like in the INTERSALT model, we tried to include potassium as a variable. Its inclusion led to slightly improved results, but due to practical drawbacks, such as higher laboratory costs and less frequent routine measurements, we developed a formula without it.

The decision to test urea was motivated by its correlations to protein intake [44] and finally to urinary sodium excretion [45]; correlations confirmed by the strong performance of SAMU.

To evaluate the benefit of using a nocturnal timed urine collection instead of the first morning urine to assess at the same time the hourly creatinine excretion, we tested targeted models, further improving the accuracy.

In summary, our study shows that the Swiss anthropometric model (SAM), the Swiss anthropometric model with potassium (SAMK), the Swiss anthropometric model with urea (SAMU), and the Swiss anthropometric model with urea and potassium (SAMUK), are all reliable alternatives to the estimation equations traditionally used. These newly developed models, still in need of external validation but showing promising performance, could be useful in both the clinical setting and at the population level. Even though the anthropometric model with urea shows the best performance, considering the modest benefit, for convenience and cost savings, we recommend using the model based exclusively on urinary sodium and creatinine in clinical settings.

This study has some limitations that should be mentioned. The external applicability may be limited due to the fact that the models were built using a Swiss population for both estimating urinary creatinine excretion and calculating the variables, and that sodium consumption is population-specific. The percentage of subjects with hypertension and chronic kidney disease was recorded but, given the size of the sample, no subgroup analyses were performed to verify the validity of the estimates within them. The same applies to therapy with drugs that may affect sodium excretion. Assuming that the subjects were in a steady state with regard to sodium excretion, we did not exclude those treated with diuretics and renin–angiotensin–aldosterone system (RAAS) inhibitors. Furthermore, estimating salt and water intake using questionnaires could have further increased the accuracy of the daily salt intake prediction, but this was not the aim of the study.

Strengths include the use of data obtained from an independent epidemiological study referring to the same population to estimate urinary creatinine excretion, and the choice of the Swiss population, known for its multi-cultural and multilingual characteristics and for being representative of the Central European population. Last but not least, the study explored the role of urea as a predictor and the added benefit of using a timed nocturnal urinary collection.

## 5. Conclusions

Based on a formula to estimate urinary creatinine excretion from an independent epidemiological study referring to the same Swiss/Central European population, and by rigorously selecting the data used to construct the models, it was possible to build reliable predictive formulae for 24 h urinary sodium excretion using first morning urine samples. Their performance is compatible with clinical use in the adult European population. Considering the modest benefit of adding potassium and urea, we recommend using the model based exclusively on urinary sodium and creatinine to achieve convenience and cost savings.

## Figures and Tables

**Table 1:** Demographic, anthropometric, and biochemical characteristics of the study population (*n* = 811), stratified by training (*n* = 574) and test set (*n* = 237).

Variables	Total (*n* = 811)	Training (*n* = 574, 70.8%)	Test (*n* = 237, 29.2%)	*p*-Value
BMI (kg/m^2^)	25.21 ± 4.4 [24.5 (22.2–27.5)]	25.25 ± 4.44 [24.5 (22.2–27.7)]	25.14 ± 4.31 [24.6 (22.1–27.3)]	0.813
Sex, *n* (%)				
Male	352 (43.4)	325 (56.6)	135 (57.0)	0.983
Female	459 (56.6)	249 (43.4)	102 (43.0)	
Weight (kg)	71.97 ± 15.8 [71.0 (60.0–81.0)]	72.26 ± 15.94 [71.0 (60.0–81.0)]	71.25 ± 15.46 [70.0 (60.0–80.0)]	0.431
Height (cm)	168.51 ± 9.95 [167.94 (161.09–175.12)]	168.78 ± 10.33 [167.94 (161.11–175.96)]	167.86 ± 8.95 [167.94 (160.96–173.95)]	0.349
Age (years)	51.19 ± 13.7 [51.0 (42.0–60.0)]	50.87 ± 13.9 [51.0 (42.0–60.0)]	51.96 ± 13.21 [51.0 (43.0–61.0)]	0.310
Expected creatinine excretion, 24 h Forni (mmol) [20]	12.04 ± 3.57 [11.05 (9.22–14.92)]	12.1 ± 3.66 [11.08 (9.2–15.17)]	11.87 ± 3.36 [10.98 (9.27–14.53)]	0.592
Observed creatinine excretion, 24 h (mmol)	12.46 ± 3.81 [11.83 (9.41–15.15)]	12.45 ± 3.91 [11.76 (9.25–15.36)]	12.47 ± 3.55 [11.84 (9.85–14.92)]	0.677
Expected creatinine excretion, 24 h Kawasaki (mmol)	14.21 ± 2.94 [14.15 (12.05–16.01)]	14.3 ± 3.03 [14.37 (12.03–16.04)]	13.98 ± 2.72 [13.79 (12.09–15.76)]	0.185
Expected creatinine excretion, 24 h Tanaka (mmol)	12.75 ± 3.16 [12.5 (10.37–14.76)]	12.84 ± 3.22 [12.53 (10.39–14.88)]	12.55 ± 3.0 [12.29 (10.3–14.43)]	0.322
Urine volume, 24 h (L)	1.81 ± 0.78 [1.7 (1.2–2.25)]	1.82 ± 0.8 [1.7 (1.2–2.25)]	1.77 ± 0.74 [1.6 (1.2–2.1)]	0.494
Urine volume, day (L)	1.27 ± 0.66 [1.1 (0.8–1.6)]	1.27 ± 0.67 [1.15 (0.8–1.64)]	1.26 ± 0.64 [1.1 (0.8–1.6)]	0.980
Urine volume, night (L)	0.54 ± 0.29 [0.5 (0.35–0.7)]	0.55 ± 0.29 [0.5 (0.35–0.7)]	0.52 ± 0.27 [0.45 (0.35–0.6)]	0.137
Na concentration, day (mmol/L)	89.94 ± 50.19 [79.0 (52.0–121.0)]	89.08 ± 51.16 [77.0 (50.0–120.0)]	92.0 ± 47.82 [82.0 (55.0–123.0)]	0.207
Na concentration, night (mmol/L)	90.49 ± 45.4 [82.0 (54.0–121.0)]	89.56 ± 45.19 [81.0 (54.0–118.0)]	92.72 ± 45.94 [82.0 (55.0–125.0)]	0.394
Creatinine concentration, day (mmol/L)	8.24 ± 4.85 [7.0 (4.6–10.8)]	8.21 ± 4.88 [7.0 (4.53–10.8)]	8.3 ± 4.78 [7.1 (4.8–11.0)]	0.676
Creatinine concentration, night (mmol/L)	9.75 ± 5.43 [8.4 (5.4–13.1)]	9.74 ± 5.62 [8.35 (5.3–13.08)]	9.78 ± 4.95 [8.4 (5.7–13.4)]	0.406
Creatinine excretion, day (mmol)	8.16 ± 2.78 [7.76 (6.0–9.98)]	8.14 ± 2.83 [7.71 (5.96–9.96)]	8.23 ± 2.67 [7.9 (6.24–9.98)]	0.516
Creatinine excretion, night (mmol)	4.29 ± 1.73 [4.0 (3.08–5.12)]	4.32 ± 1.77 [4.05 (3.08–5.12)]	4.24 ± 1.62 [3.87 (3.08–5.11)]	0.603
Night hours (h)	8.29 ± 2.09 [8.07 (7.07–9.24)]	8.33 ± 2.07 [8.11 (7.13–9.29)]	8.21 ± 2.14 [7.96 (6.9–9.18)]	0.305
Hourly creatinine excretion (mmol/h)	0.52 ± 0.16 [0.49 (0.39–0.63)]	0.52 ± 0.16 [0.49 (0.39–0.64)]	0.52 ± 0.15 [0.49 (0.41–0.62)]	0.677
Hourly Na excretion (mmol/h)	5.34 ± 2.75 [4.75 (3.43–6.79)]	5.33 ± 2.79 [4.79 (3.36–6.66)]	5.34 ± 2.66 [4.71 (3.56–6.93)]	0.809
Na concentration, night/creatinine concentration, night	10.57 ± 5.0 [9.55 (7.06–12.92)]	10.64 ± 5.2 [9.61 (7.07–13.01)]	10.39 ± 4.49 [9.46 (7.03–12.86)]	0.895
Na excretion, 24 h (mmol)	139.14 ± 56.05 [133.7 (96.0–172.9)]	138.26 ± 56.47 [132.32 (96.05–170.69)]	141.29 ± 55.08 [135.2 (96.0–175.5)]	0.468
Na excretion, day (mmol)	95.31 ± 43.97 [90.25 (63.2–120.0)]	94.11 ± 43.57 [88.8 (63.2–118.61)]	98.22 ± 44.9 [92.0 (63.2–126.35)]	0.294
Na excretion, night (mmol)	43.83 ± 24.51 [38.5 (26.8–55.58)]	44.15 ± 25.36 [38.5 (26.6–55.5)]	43.08 ± 22.35 [38.4 (27.2–56.4)]	0.991
K excretion, night (mmol/L)	28.9 ± 15.95 [26.0 (17.0–37.0)]	28.51 ± 16.38 [25.0 (16.0–37.0)]	29.81 ± 14.89 [27.0 (19.0–38.0)]	0.083
Urea excretion, night (mmol/L)	281.18 ± 142.63 [250.55 (166.78–375.38)]	280.13 ± 145.02 [250.2 (162.1–381.8)]	283.64 ± 137.14 [251.05 (173.38–371.18)]	0.563
K, night/creatinine, night	3.35 ± 1.46 [3.1 (2.25–4.09)]	3.34 ± 1.48 [3.08 (2.31–4.07)]	3.35 ± 1.43 [3.19 (2.17–4.14)]	0.745
Urea, night/creatinine night	0.53 ± 0.52 [0.37 (0.2–0.69)]	0.55 ± 0.55 [0.37 (0.2–0.71)]	0.49 ± 0.46 [0.37 (0.2–0.64)]	0.539
Predicted Na excretion, Kawasaki 24 h (mmol)	64.9 ± 27.87 [58.94 (44.61–81.59)]	65.83 ± 29.0 [59.31 (44.71–82.75)]	62.67 ± 24.83 [57.36 (43.54–76.38)]	0.314
Predicted Na excretion, Tanaka 24 h (mmol)	65.62 ± 22.8 [61.41 (49.06–79.29)]	66.33 ± 23.69 [61.59 (49.06–80.29)]	63.91 ± 20.42 [61.1 (49.06–75.12)]	0.337
Predicted Na excretion, INTERSALT 24 h (mmol)	97.06 ± 24.95 [93.0 (79.26–114.68)]	96.95 ± 24.96 [92.73 (79.27–114.74)]	97.33 ± 24.98 [94.05 (79.23–114.52)]	0.971

Data are reported as mean ± standard deviation [median (interquartile range)]. Differences between groups were assessed using the Mann–Whitney U test for continuous variables and the chi-square test for categorical variables.

**Table 2:** Validation results of the three predictive models using first morning urine samples.

Model	R^2^	RMSE	Mean Difference	Relative Error (Mean ± SD)	Error > 40% *
Swiss anthropometric model with potassium (SAMK)	0.52	38.06	−5.5 (37.99)	3.65 ± 28.22	52 (21.94%)
Swiss anthropometric model (SAM)	0.53	37.99	−4.23 (37.91)	4.49 ± 28.52	38 (16.03%)
Swiss anthropometric model with urea and potassium (SAMUK)	0.54	37.38	−2.86 (37.31)	5.54 ± 28.46	51 (21.54%)
Swiss anthropometric model with urea (SAMU)	0.53	37.48	−5.01 (37.42)	3.80 ± 27.98	49 (20.68%)

* Number of patients with a relative error above 40%—reported metrics include the coefficient of determination (R^2^), root mean squared error (RMSE), mean bias (difference between predicted and observed values), relative error (mean ± SD), and proportion of individuals with a relative error greater than 40%.

**Table 3:** Validation results of the three predictive models using timed nocturnal urine samples.

Model	R^2^	RMSE	Mean Difference	Relative Error (Mean ± SD)	Error > 40% *
Swiss anthropometric model with potassium (SAMK)	0.57	36.12	10.37 (36.04)	15.9 ± 30.22	68 (28.69%)
Swiss anthropometric model (SAM)	0.58	35.92	−3.88 (35.84)	4.03 ± 26.65	29 (12.24%)
Swiss anthropometric model with urea and potassium (SAMUK)	0.58	35.52	−2.76 (35.45)	4.80 ± 26.58	50 (21.10%)
Swiss anthropometric model with urea (SAMU)	0.58	35.54	−2.60 (35.48)	4.95 ± 26.55	51 (21.52%)

* Number of patients with a relative error above 40%. Reported metrics include the coefficient of determination (R^2^), root mean squared error (RMSE), mean bias (difference between predicted and observed values), relative error (mean ± SD), and proportion of individuals with a relative error greater than 40%.

**Table 4:** Area under the receiver operating characteristic (ROC) curve (AUC) for the four predictive models with first morning urine and standard estimation formulae (Kawasaki, Tanaka, and INTERSALT) at sodium excretion thresholds of 85, 150, and 200 mmol/day.

Model	AUC (95% CI)
Cut-off: 150 mmol	
SAMUK	0.88 (0.84–0.93)
SAMU	0.89 (0.84–0.93)
SAMK	0.88 (0.84–0.93)
SAM	0.88 (0.83–0.93)
Tanaka	0.64 (0.57–0.72)
Kawasaki	0.67 (0.60–0.74)
INTERSALT	0.75 (0.68–0.81)
Cut-off: 85 mmol	
SAMUK	0.87 (0.81–0.92)
SAMU	0.87 (0.81–0.92)
SAMK	0.87 (0.82–0.92)
SAM	0.87 (0.82–0.92)
Tanaka	0.70 (0.61–0.78)
Kawasaki	0.72 (0.64–0.81)
INTERSALT	0.72 (0.63–0.81)
Cut-off: 200 mmol	
SAMUK	0.92 (0.87–0.96)
SAMU	0.92 (0.88–0.97)
SAMK	0.92 (0.87–0.96)
SAM	0.92 (0.87–0.96)
Tanaka	0.71 (0.62–0.81)
Kawasaki	0.74 (0.65–0.83)
INTERSALT	0.74 (0.65–0.82)

## Data Availability

The original contributions presented in this study are included in the article and Supplementary Materials. Further inquiries can be directed to the corresponding author.

## Supplementary Materials

The following supporting information can be downloaded at: https://www.mdpi.com/article/10.3390/nu17203284/s1, Table S1: Training set multivariate linear regression models based on first morning urine samples; Table S2: Training set multivariate linear regression models based on timed nocturnal urine samples.

## Abbreviations

The following abbreviations are used in this manuscript:

BP	Blood pressure
CVD	Cardiovascular disease
SAM	Swiss anthropometric model
SAMK	Swiss anthropometric model with potassium
SAMUK	Swiss anthropometric model with urea and potassium
SAMU	Swiss anthropometric model with urea
