# Supplementary material for: New Anthropometry-Based Formulae to Predict 24 h Sodium Excretion from Spot Urine
Source: Nutrients. 2025 Oct 20;17(20):3284. doi: 10.3390/nu17203284 (PMC12566651; doi:10.3390/nu17203284)
Supplement: Supplementary file 1 [file nutrients-17-03284-s001.zip › nutrients-3886448-supplementary.pdf]

Table S1 – Training set multivariate linear regression models based on first morning urine samples

	SAMK	SAM	SAMUK	SAMU
Na night/Creat night · Estimated Creatinine (mmol/L)	0.543 (p≤0.001) [0.483; 0.602]	0.569 (p≤0.001) [0.515; 0.623]	0.557 (p≤0.001) [0.497; 0.617]	0.559 (p≤0.001) [0.499; 0.619]
K night (mmol/L)	0.054 (p=0.630) [-0.165; 0.272]		-0.186 (p=0.186) [-0.462; 0.090]	
Urea night (mmol/L)			0.046 (p=0.006) [0.013; 0.079]	0.033 (p=0.014) [0.007; 0.058]
Weight (kg)	0.519 (p≤0.001) [0.243; 0.796]	0.470 (p≤0.001) [0.210; 0.730]	0.443 (p=0.002) [0.163; 0.723]	0.446 (p=0.002) [0.166; 0.726]
Height (cm)	-0.055 (p=0.800) [-0.485; 0.374]	-0.015 (p=0.942) [-0.415; 0.386]	-0.091 (p=0.677) [-0.518; 0.337]	-0.108 (p=0.620) [-0.535; 0.319]
Age (years)	-0.520 (p≤0.0019 [-0.777; -0.264]	-0.474 (p≤0.001) [-0.711; -0.236]	-0.439 (p=0.001) [-0.700; -0.178]	-0.467 (p≤0.001) [-0.725; -0.209]
R2	0.51	0.52	0.52	0.51

SAMK = Swiss anthropometric model including potassium; SAM = Swiss anthropometric model; SAMUK = Swiss anthropometric model with urea and potassium; SAMU = Swiss anthropometric model with urea without potassium. Each cell reports the β coefficient, p-value, and 95% confidence interval (CI) derived from multivariate linear regression models in the training set.

Table S2 – Training set multivariate linear regression models based on timed nocturnal urine samples

	SAMK	SAM	SAMUK	SAMU
Hourly Na concentration (mmol/L) · 24h	0.580 (p ≤ 0.001) [0.526; 0.634]	0.597 (p≤0.001) [0.548; 0.646]	0.590 (p≤0.001) [0.536; 0.644]	0.592 (p≤0.001) [0.538; 0.646]
K night (mmol/L)	0.033 (p = 0.751) [-0.170; 0.235]		-0.181 (p = 0.168) [-0.438; 0.076]	
Urea night (mmol/L)			0.041 (p = 0.009) [0.010; 0.071]	0.027 (p = 0.025) [0.004; 0.051]
Weight (kg)	0.456 (p=0.001) [0.198; 0.713]	0.409 (p=0.001) [0.166; 0.652]	0.391 (p=0.003) [0.131; 0.652]	0.394 (p=0.003) [0.134; 0.655]
Height (cm)	-0.037 (p=0.856) [-0.435; 0.361]	0.026 (p=0.891) [-0.347; 0.399]	-0.063 (p=0.757) [-0.459; 0.334]	-0.079 (p=0.696) [-0.475; 0.317]
Age (years)	-0.514 (p≤0.001) [-0.752; -0.275]	-0.493 (p≤0.001) [-0.715; -0.272]	-0.441 (p≤0.001) [0.685; -0.198]	-0.469 (p≤0.001 [-0.709; -0.228]
R2	0.57	0.58	0.58	0.58

Legend: SAMK = Swiss anthropometric model including potassium; SAM = Swiss anthropometric model; SAMUK = Swiss anthropometric model with urea and potassium; SAMU = Swiss anthropometric model with urea without potassium. Each cell reports the β coefficient, p-value, and 95% confidence interval (CI) derived from multivariate linear regression models in the training set.
